# Spatiotemporal Surface Temperature Measurements Resolving Flame-Wall Interactions of Lean H_2_-Air and CH_4_-Air Flames Using Phosphor Thermometry

**DOI:** 10.1007/s10494-024-00571-1

**Published:** 2024-08-05

**Authors:** Anthony O. Ojo, Abhijit Padhiary, Brian Peterson

**Affiliations:** Institute of Multiscale Thermofluids, School of Engineering, The University of Edinburgh, Edinburgh, UK

**Keywords:** Flame-wall interaction, Phosphor thermometry, Wall temperature, Hydrogen, Thermodiffusive instabilities

## Abstract

Spatiotemporal wall temperature (T_wall_) distributions resulting from flame-wall interactions of lean H_2_-air and CH_4_-air flames are measured using phosphor thermometry. Such measurements are important to understand transient heat transfer and wall heat flux associated with various flame features. This is particularly true for hydrogen, which can exhibit a range of unique flame features associated with combustion instabilities. Experiments are performed within a two-wall passage, in an optically accessible chamber. The phosphor ScVO_4_:Bi^3+^ is used to measure T_wall_ in a 22 × 22 mm^2^ region with 180 µm/pixel resolution and repetition rate of 1 kHz. Chemiluminescence imaging is combined with phosphor thermometry to correlate the spatiotemporal dynamics of the flame with the heat signatures imposed on the wall. Measurements are performed for lean H_2_-air flames with equivalence ratio Φ = 0.56 and compared to CH_4_-air flames with Φ = 1. T_wall_ signatures for H_2_-air Φ = 0.56 exhibit alternating high and low-temperature vertical streaks associated with finger-like flame structures, while CH_4_-air flames exhibit larger scale wrinkling with identifiable crest/cusp regions that exhibit higher/lower wall temperatures, respectively. The underlying differences in flame morphology and T_wall_ distributions observed between the CH_4_-air and lean H_2_-air mixtures are attributed to the differences in their Lewis number (CH_4_-air Φ = 1: Le = 0.94; H_2_-air Φ = 0.56: Le = 0.39). Findings are presented at two different passage spacings to study the increased wall heat loss with larger surface-area-to-volume ratios. Additional experiments are conducted for H_2_-air mixtures with Φ = 0.45, where flame propagation was slower and was more suitable to resolve the wall heat signatures associated with thermodiffusive instabilities. These unstable flame features impose similar wall heat fluxes as flames with 2–3 times greater flame power. In this study, these flame instabilities occur within a small space/time domain, but demonstrate the capability to impose appreciable heat fluxes on surfaces.

**Supplementary Information:**

The online version contains supplementary material available at 10.1007/s10494-024-00571-1.

## Introduction

Flame-wall interaction (FWI) is an important topic within gas turbine and internal combustion (IC) engines, where a flame is bounded by solid surfaces. As a flame interacts with the wall, it imposes a significant heat flux on the wall, which subsequently weakens the flame. This mutual interaction defines the flame quenching distance from the wall, the near-wall emission formation, and the thermal fatigue imposed on the chamber walls (Dreizler and Böhm 2015). Modern engines are designed to be smaller and operate with higher power densities, as these conditions are conducive for reducing fuel consumption and CO_2_ emissions (Szybist et al. 2021; Leach et al. 2020). However, in these downsized combustors, the surface-area to volume ratio increases such that the flame is more exposed to walls, and higher pressures are such that more chemical reactions occur near surfaces (Johe et al. 2022). These trends are making FWI an increasingly important topic within combustion research.

Another prevailing topic in combustion research is the transition to low-carbon and zero-carbon fuels (Leach et al. 2020; Masri 2021; Onorati et al. 2022). In this area, hydrogen (H_2_) has received significant attention as a potential fuel that can help decarbonize transportation and power generation sectors (Onorati et al. 2022; Taamallah et al. 2015; Verhelst and Wallner 2009). Substantial research efforts have demonstrated the successful implementation of baseline IC engines converted to operate with hydrogen fuel (Leite et al. 2023a; 2023b) and the use of hydrogen enrichment with traditional jet fuels in model gas turbine combustors (Agostinelli et al. 2022; Aniello et al. 2022; Chterev and Boxx 2021; Miniero et al. 2023). Such research has yielded promising results in terms of hydrogen feasibility, while achieving low carbon, soot, and nitric oxide emissions.

Although hydrogen has emerged as a promising fuel for reducing carbon emissions, several aspects need to be addressed regarding its implementation. Among these aspects are FWI and heat transfer to surfaces. Hydrogen exhibits high heating values, high adiabatic flame temperatures, and has one of the highest flame speeds among the available fuels (Konnov et al. 2018). Flame quenching distance is inversely proportional to flame temperature and flame speed (Ferguson and Keck 1977; Lavoie 1978), such that wall heat fluxes are often greater with hydrogen. Lean mixtures of hydrogen can reduce flame temperature and flame speed, but lean H_2_ flames are subject to thermodiffusive instabilities. These combustion instabilities occur when the Lewis number (Le) is low such that for a wrinkled lean hydrogen flame the local mass flux of H_2_ into the reaction zone is greater than the heat flux out of the reaction zone. This preferential diffusion promotes local H_2_ enrichment along a corrugated flame surface (Berger et al. 2019), which increases the local flame speed and the local flame temperatures (Frouzakis et al. 2015, Berger et al. 2022; Howarth and Aspden 2022; Howarth et al. 2023). These higher temperature flame regions can cause concerns of high wall heat fluxes and thermal fatigue of surfaces.

Wall temperature is a key component to understand FWI and wall heat fluxes. Thermocouples, heat flux gauges, and phosphor thermometry are common experimental techniques used to measure wall temperature during FWI (e.g., Padhiary et al. 2023; Boust et al. 2007; Aldén et al. 2011). In terms of hydrogen FWI, experimental measurements of wall temperature are sparse, with only a few exceptions reported in the literature (Guiberti et al. 2015; Feuk et al. 2022; Nau et al. 2023). Direct numerical simulations (DNS) have played an instrumental role in understanding FWI for H_2_ flames (e.g., Owston et al. 2007; Gruber et al. 2010; Mari et al. 2016; Lai et al. 2022; Zhao et al. 2022; Zhu et al. 2024). The majority of DNS studies employ isothermal or adiabatic wall boundary conditions. While these simplified conditions provide the means to perform DNS in a reasonable manner, these conditions can deviate from true FWI conditions. One-dimensional simulations employing conjugate heat transfer (CHT) have revealed significant differences in FWI characteristics compared to isothermal and adiabatic conditions (Mari et al. 2016). To understand the effects of wall heat transfer, Agostinelli et al. (2022) employed CHT in Large Eddy Simulations (LES) of a swirl burner to resolve the increased bluff-body surface temperature induced by shorter flame lengths from H_2_ enrichment. The higher wall temperature led to increased heat transfer to the gaseous fluid, which modified the flame/flow dynamics and was argued to play an underlying role in the observed thermoacoustic instabilities in experiments. These findings reveal the important effects of wall heat transfer associated with H_2_ flames.

The wide-spread use of hydrogen in gas turbines and IC engines is still in development, and with this, hydrogen combustion heat transfer models are also still under development (Demuynck et al. 2011; Michl et al. 2016). The lack of experimental data on wall temperature and boundary conditions impedes the development of such models. Consequently, most engine combustion simulations are not fully predictive with respect to wall heat transfer. Most engine-related wall models utilize empirical constants that are adjusted to match experimental conditions, such as gas pressure or heat release (Rakopoulos et al. 2010). Recent studies employing hydrogen in IC engines revealed that wall model empirical constants are appreciably different for hydrogen than hydrocarbon fuels (Leite et al. 2023a, b). Detailed measurements of wall temperature during hydrogen FWI are needed to improve the predictive capability of wall models.

Within gas turbine and IC engines, flames impinge onto individual chamber surfaces, as well as within two-wall engine passages (e.g., annular slots, flame holders, crevices, etc.). Heat transfer and flame quenching are often most severe in two-wall passages where the flame is more exposed to walls. The heat transfer in these passages can affect acoustic instabilities in gas turbine combustors (Ketelheun et al. 2013; Mejia et al. 2015; Agostinelli et al. 2022) and the unburned fuel emissions and temperature stratification in IC engines (Alkidas 1999; Dronniou and Dec 2012; Peterson et al. 2013, 2014). Measuring wall temperature and FWI in two-wall passages is often not performed due to limited measurement access. Numerical simulations have highlighted the need for detailed wall temperature measurements in various narrow passage configurations to validate simulations and better understand the underlying FWI physics (Janas et al. 2015; Agostinelli et al. 2022). Such statements are further emphasized for hydrogen flames, which can penetrate further into narrow passages owing to their high flame speed and smaller flame thicknesses.

In this work, we experimentally measure wall temperatures (T_wall_) associated with FWI of hydrogen within a two-wall narrow passage. Phosphor thermometry is used to obtain spatially resolved 2D T_wall_ measurements during FWI. The thermographic phosphor used is bismuth-doped scandium vanadate (ScVO_4_:Bi^3+^), which offers high temperature sensitivity and measurement precision (Abram et al. 2020; Ojo et al. 2022). Experiments are performed in a fixed-volume chamber (FVC) designed to emulate a simplified piston engine geometry and is equipped with an optically accessible two-wall passage. Phosphor thermometry is performed simultaneously with chemiluminescence imaging to understand the spatiotemporal features of T_wall_ and the flame front. Experiments are performed with lean H_2_-air mixtures (equivalence ratio, Ф = 0.56), as well as stoichiometric CH_4_-air mixtures. Findings from CH_4_ flames are presented to highlight the unique spatiotemporal T_wall_ signatures associated with the intrinsically different flame structure between H_2_ and CH_4_ flames. Findings are presented for two passage spacings to study the wall heat transfer as the surface-are to volume ratio increases. This work concludes by presenting additional experiments with leaner H_2_-air mixtures (Φ = 0.45) to investigate wall heat flux signatures associated with thermodiffusive instabilities. These unstable flame features are shown to impose similar magnitudes of wall heat flux as flames with 2–3 times greater flame power.

## Experimental

### Fixed Volume Chamber (FVC)

**Fig. 1:**
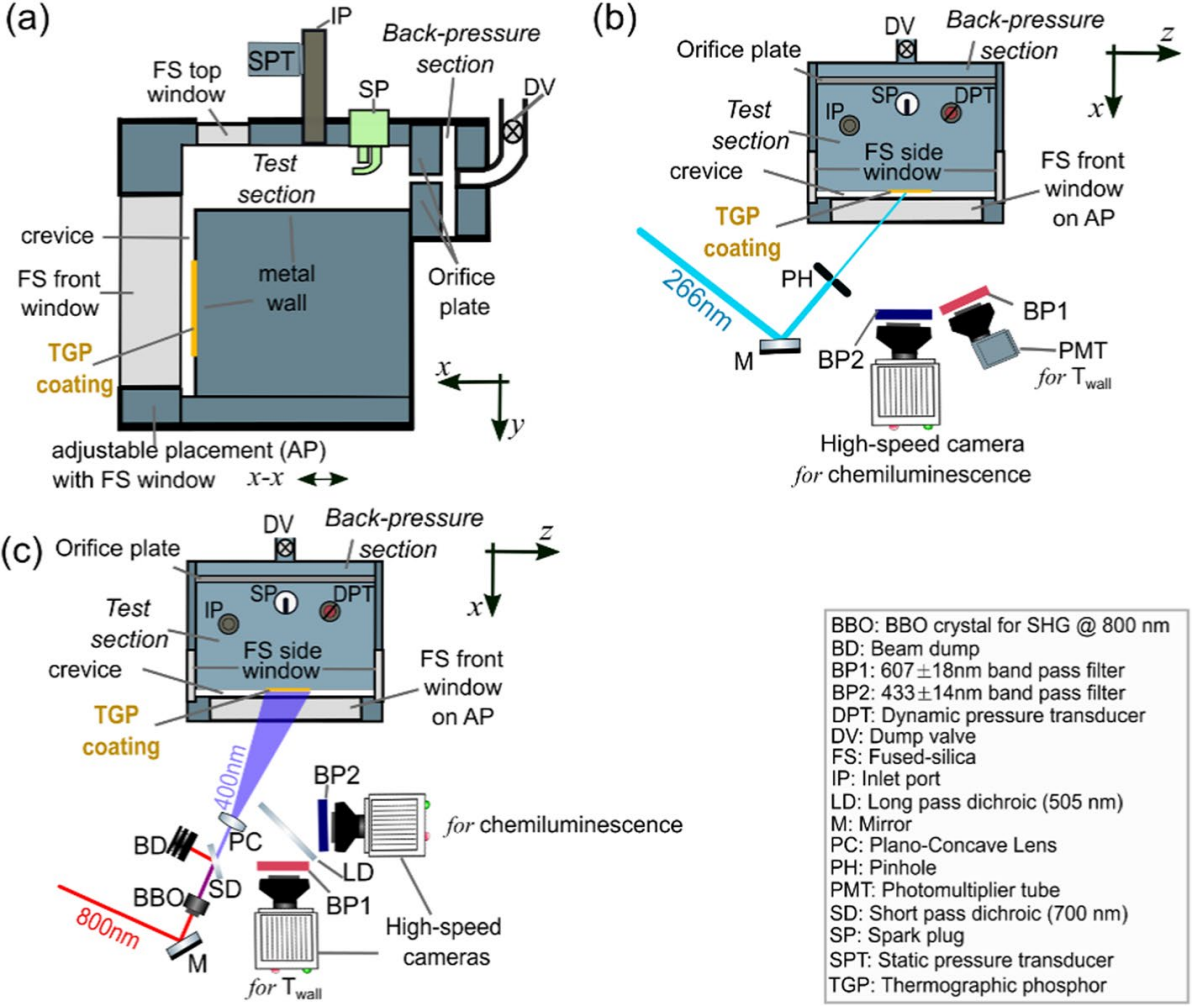
**a** schematic of the FVC, **b** experimental setup for 0D phosphor thermometry and flame imaging, **c** experimental setup for 2D phosphor thermometry and flame imaging

Experiments were performed in an optically accessible fixed volume chamber (FVC) dedicated for FWI and heat transfer studies. A schematic of the FVC is shown in Fig. 1. The FVC features a test section (150 cm^3^) and a back pressure section (6 cm^3^). These sections are separated by a 6 mm thick orifice plate with 81 equidistant holes each with 0.5 mm diameter. The test section emulates a simplified IC engine geometry at top-dead center. At the far end of the test section, a two-walled crevice region exists, which is designed to emulate an enlarged piston crevice. Fused silica windows are used to provide optical access into the test section and crevice region. All metal components of the FVC are comprised of 304 stainless-steel.

The chamber operation has been described by Escofet-Martin et al. (2021) and Ojo et al. (2021) and is briefly described here. The contents inside the FVC are initially evacuated to 20 mbar. A homogenous fuel–air mixture is then introduced into the chamber until an initial pressure of 2 bar is reached. The fuel–air mixture is ignited by the spark plug. Heat release initiates an exponential pressure rise as the flame propagates towards the opposite end of the chamber before entering the crevice region. At a preselected chamber pressure, a dump-valve with 8 ms response time is activated to evacuate the gas inside the chamber. The exhaust flow exiting the FVC is choked by the orifice plate to create an exponential pressure decay that closely mirrors the exponential pressure rise. The chamber operation is intended to simulate a pressure time history similar to that of an IC engine albeit at lower pressures.

Similar to our previous works (Ojo et al. 2022, 2023), this work focuses on T_wall_ measurements and flame front imaging within the crevice passage of the FVC. The crevice region is 70 mm deep ($\Delta y$) and 158 mm wide ($\Delta z$). The crevice is characterized by two walls: one is the front fused silica window surface, and the other is the 304 stainless steel wall (see Fig. 1a). These walls are not externally cooled or heated. The distance between the walls is referred to as the crevice spacing (CS). The placement of the front window can be adjusted to select a CS between 0.5–5.0 mm (Ojo et al. 2022). The front window with size of 60 $\times$ 110 mm^2^ ($\Delta y\times \Delta z$) provides optical access to the majority of the crevice region, as well as 2 mm ($\Delta y$) above the crevice within the test section.

In this work, three different homogeneous fuel–air mixtures were investigated: (1) a H_2_-air mixture with equivalence ratio of Ф = 0.56, (2) a CH_4_-air mixture with Ф = 1.0, and (3) a H_2_-air mixture with Ф = 0.45. The first two mixtures were chosen in an attempt to provide flames with a comparable flame power. Flame power (${Q}_{\Sigma }$) represents a laminar heat release reference, and is often used to normalize the wall heat flux in FWI studies (Poinsot and Veynante 2005; Dreizler and Böhm 2015). ${Q}_{\Sigma }$ is defined as:

1 $$Q_{{\Sigma }} = \rho_{u} S_{L} C_{p} \left( {T_{ad} - T_{u} } \right)$$

where ${\rho }_{u}$ is the unburned gas density, ${S}_{L}$ is the unstretched laminar flame speed, ${C}_{p}$ is the heat capacity of the unburned mixture, ${T}_{ad}$ is the adiabatic flame temperature, and ${T}_{u}$ is the unburned gas temperature.

Table 1 reports the operating conditions explored in this work and their corresponding ${Q}_{\Sigma }$, ${S}_{L}$, ${T}_{ad}$, and Lewis number (Le). These properties were calculated from Cantera (1D flame simulation), which utilized the GRI 3.0 Mechanism (Smith et al. 2011). Properties are reported for time-averaged pressure/temperature conditions when the flame is within the measurement region of interest (ROI). The fast heat release of hydrogen made it challenging to control the chamber pressure during the time of FWI at the measurement location. The pressure affects the value of ${S}_{L}$, which consequently affects the value of ${Q}_{\Sigma }$. A H_2_-air mixture with Ф = 0.56 provided repeatable chamber operation, but yielded a flame power 45% higher than the flame power for CH_4_-air Ф = 1.0. Leaner H_2_-air mixtures produced larger variability in the flame arrival time at the measurement ROI. The variability in the flame arrival produced a variability of the chamber pressure, which made it difficult to control variables such as ${S}_{L}$ and ${Q}_{\Sigma }$. It was decided that a repeatable operating condition was preferred over ensuring an iso-condition between the two mixtures. As such, the H_2_-air Ф = 0.56 operation was chosen for qualitative comparison to the CH_4_-air Ф = 1.0 operation. Given that the mixtures do not have an iso-condition (i.e., similar value of ${Q}_{\Sigma }$, ${S}_{L}$, or ${T}_{ad}$), comparison of the findings between these mixtures is performed with caution.

**Table 1:** Operating conditions of experiments performed in the FVC

Fuel–air mixture	CS	Time avg. pressure when flame is in ROI (bar)	${Q}_{\Sigma }$(MW/m^2^)	${S}_{L}$(m/s)	${T}_{ad}$(K)	Effective Lewis number^a^
H_2_-air (Ф = 0.56)	2.0 mm	6.0 bar	6.2	0.92	1895	0.39
CH_4_-air (Ф = 1.0)	2.0 mm	4.9 bar	4.3	0.43	2374	0.94
H_2_-air (Ф = 0.56)	1.2 mm	6.0 bar	6.2	0.92	1895	0.39
CH_4_-air (Ф = 1.0)	1.2 mm	4.9 bar	4.3	0.43	2374	0.94
H_2_-air (Ф = 0.45)	1.2 mm	5.0 bar	1.7	0.35	1708	0.36

^a^The effective Lewis number ($L{e}_{eff}$) was calculated following that of Berger et al. (2022): $L{e}_{eff}=1+\left[\left(L{e}_{E}-1\right)+\left(L{e}_{D}-1\right)A\right]/(1+\text{A})$. Subscripts $E,D$ refer to the excess and deficient species in the reactants, respectively, and $A$ is defined as $A=1+\beta (\Phi -1)$, where $\beta$ is the Zel’dovich number

The H_2_-air Ф = 0.56 mixture has a low Lewis number of 0.39, for which the flame can undergo thermodiffusive instabilities (Berger et al. 2022). However, under the H_2_-air Ф = 0.56 condition, the flame propagation is fast and it is difficult to capture a series of consecutive images of the flame passing through the ROI. Therefore, the leaner H_2_-air mixture of Ф = 0.45, shown in Table 1, was chosen to slow the H_2_ flame propagation to resolve the spatiotemporal T_wall_ distribution for a series of consecutive images as the flame propagates in the ROI. Under these conditions, it was more conducive to capture the temporal sequence of thermodiffusive instabilities associated with lean H_2_-air flames.

As shown in Table 1, experiments were performed with two different crevice spacings of 2 mm and 1.2 mm. The CS prescribes the surface-area to volume ratio (*SA/V*) in the crevice, which largely governs gas/wall heat exchange (Ojo et al. 2022). The corresponding *SA/V* values for the 2 mm and 1.2 mm spacings are 1.02 mm^−1^ and 1.69 mm^−1^, respectively. *SA/V* values for real piston engines (including optical engines) can range from 4 to 10 mm^−1^ (Heywood 1988; Cheng et al. 1993; Baum et al. 2014; Janas et al. 2015). The *SA/V* values in this work are smaller than real engines due to a larger crevice spacing, which gives a larger volume. Although *SA/V* values are not matched to that of a piston engine, this work provides fundamental measurements to demonstrate the effect of *SA/V* on the wall heat loss.

**Fig. 2:**
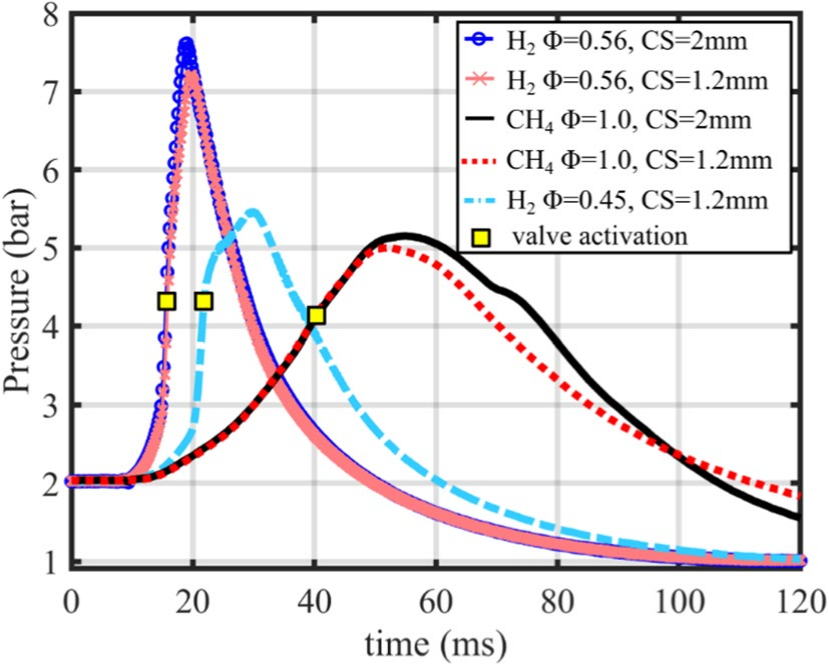
Pressure time history at each operating condition explored in this work

Figure 2 shows the pressure curves from individual experiments at each operating condition. The chamber pressures were measured using a piezo-electric pressure transducer installed in the FVC. The pressure at which the dump-valve was activated for each operating condition is shown in Fig. 2. The dump valve activation initiates the evacuation of the chamber gases, which in part influences the peak pressures and symmetry of the pressure curves. For each operation condition, the variation of the pressure curve was less than 5% from those shown in Fig. 2. The higher heat release of H_2_ is obvious from the pressure trace; the pressure rise for H_2_-air Ф = 0.56 conditions is ~ 1.37 bar/ms, while the pressure rise is ~ 0.105 bar/ms for the CH_4_-air Ф = 1.0 conditions. The peak pressures are also 2–3 bar higher for the H_2_-air Ф = 0.56 operation. The leaner H_2_-air mixture of Ф = 0.45 reaches peak pressures similar to the CH_4_-air mixture. The lower peak pressure is attributed to the leaner mixture and the timing of the dump valve relative to the flame progression.

The pressure curves at different CS reveal the influence of heat transfer on the available heat release. The pressure differences between a CS of 2.0 mm and 1.2 mm are noticeable for both the H_2_ and CH_4_ mixtures. Higher peak pressures are recorded for the 2.0 mm spacing, indicating higher heat release due to a larger volume available for gases to burn within the crevice region. The longer duration of FWI for the CH_4_-air condition yields more time for heat loss, which is largely attributed to more noticeable differences in the pressure curves at different CS.

### Phosphor Thermometry

**Fig. 3:**
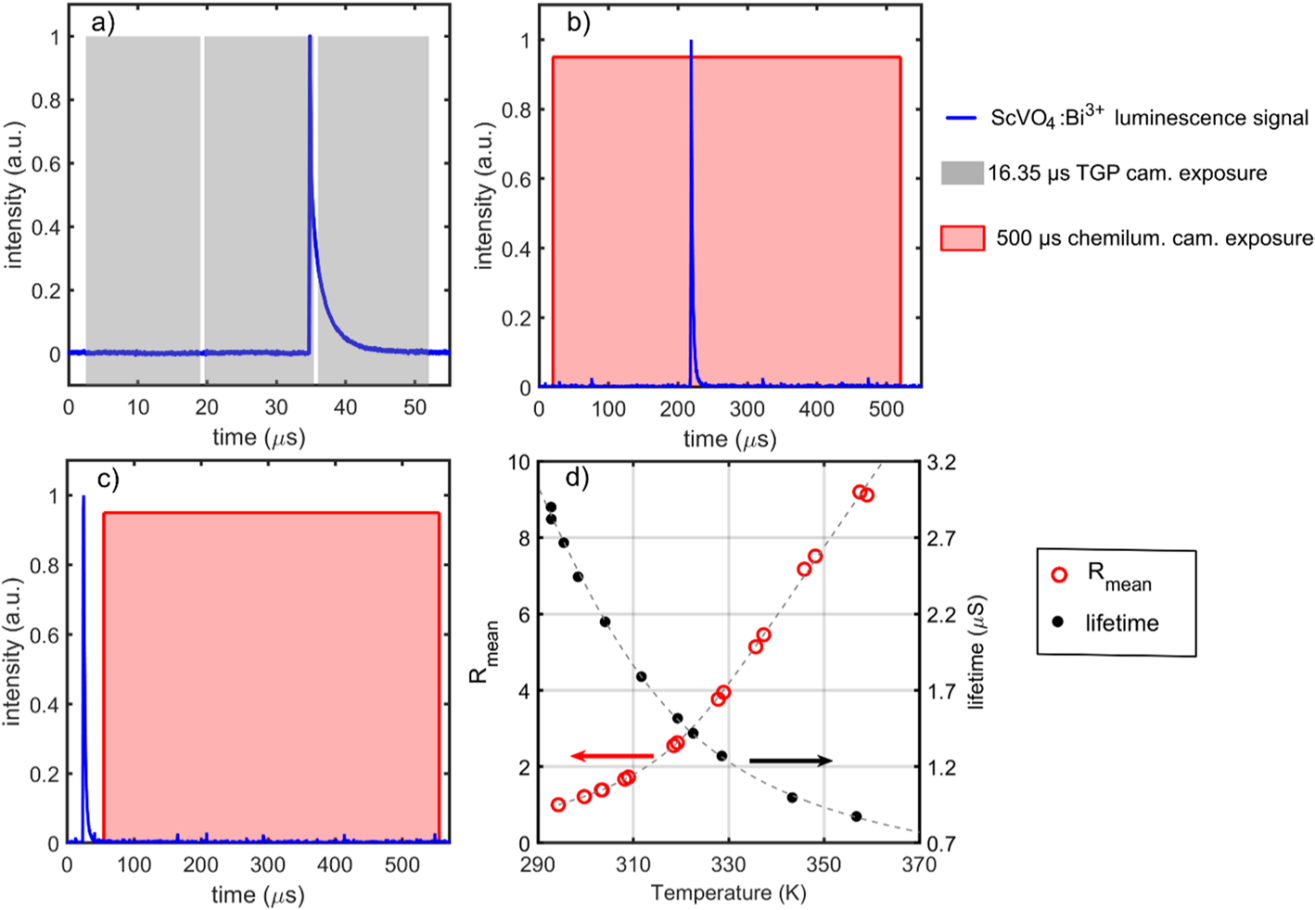
**a** Image timing sequence in relation to the phosphor decay, **b** CH* image exposure relative to the phosphor decay for CH_4_-air experiments, **c** chemiluminescence image exposure relative to the phosphor decay for H_2_-air experiments, **d** calibration curve for the 2D and 0D phosphor measurements

The phosphor used for surface thermometry was bismuth-doped (1%) scandium vanadate (ScVO_4_:Bi^3+^). ScVO_4_:Bi^3+^ features a fast phosphorescence decay with a lifetime < 3 µs at room temperature, which makes it ideal for kHz-rate measurements (Ojo et al. 2022). ScVO_4_:Bi^3+^ has a broadband emission centred around 640 nm with emission extending from ~ 400 nm to the infrared above 780 nm. Figure 3a shows an example of the phosphorescence decay signal of ScVO_4_:Bi^3+^ at room temperature. ScVO_4_:Bi^3+^ also exhibits a high temperature sensitivity, which can yield single-shot temperature precision less than 0.5 K in the temperature range of 295–335 K (Abram et al. 2020; Ojo et al. 2022, 2023). The phosphor used in this work was synthesized by Phosphor Technology Ltd, and has been used previously in FWI environments (Ojo et al. 2022). Further details of the phosphor’s luminescence properties can be found in Escofet-Martin (2022).

ScVO_4_:Bi^3+^ powder was mixed with a temperature-resistant HPC binder (ZYP coatings) and was applied using an airbrush to produce a 30 mm × 36 mm ($\Delta y\times \Delta z$) coating on the metal wall in the crevice region (see Fig. 1). The coating thickness was measured at various spatial locations using a coating thickness gauge (PCE Instruments; PCE-CT-65). The average coating thickness was $\Delta x$ = 6 ± 1 µm.

Two different experimental setups were used for phosphor thermometry. The first setup measured T_wall_ at a single location (0D) at 10 kHz repetition rate (see Fig. 1b), while the second setup measured spatially-resolved 2D T_wall_ distributions at 1 kHz repetition rate (see Fig. 1c). The former was used to better resolve the temporal evolution of T_wall_, which was not well resolved at 1 kHz for H_2_-air Ф = 0.56 conditions.

For the 0D measurements, the phosphor coating was excited by a frequency-quadrupled Nd:YAG laser (Edgewave) emitting light at 266 nm. Using a pinhole, the laser beam covered an area of about 7 mm^2^ on the phosphor coating and was placed at the central $\Delta y$ location on the phosphor coating. The laser operated at 10 kHz and had a laser energy of 0.004 mJ/pulse. A photomultiplier tube (PMT; R955HA, Hamamatsu) and a 50 mm lens were used to collect the phosphorescence decay. A 607 ± 18 nm filter was mounted to the lens to detect the peak of the ScVO_4_:Bi^3+^ emission. An oscilloscope (Tektronix MDO3054) was used to acquire the detected signals. Luminescence decays were processed on a single-shot basis to evaluate individual luminescence lifetimes. Calibration of the lifetime with temperature was performed by measuring the temperature of a phosphor-coated aluminium-block, which was heated on a hotplate up to 373 K and then allowed to cool slowly to ambient. The block was equipped with a thermocouple attached to its surface, providing a measure of the surface temperature.

For the 2D measurements, the phosphor coating was excited by a 400 nm laser beam generated from a Ti:Sapphire laser (Astrella, Coherent) emitting light at 800 nm with 35 fs pulse width. A BBO crystal was used to convert the laser to the 400 nm wavelength. It should be noted that while nanosecond excitation of thermographic phosphors is conventional for laser-based phosphor thermometry applications, femtosecond excitation of ScVO_4_:Bi^3+^ does not impede the exploitation of its emission or the lifetime for thermometry (Ojo et al. 2023, Escofet-Martin et al. 2022)*.* A − 75 mm lens was used to expand the 400 nm laser beam to a diameter of ~ 35 mm onto the phosphor coating. The laser operated at 1 kHz and had a laser energy of 0.15 mJ/pulse at 400 nm. A high-speed camera (VEO 710L, Phantom) was used to image the phosphorescence from the laser excitation. A 50 mm Nikon lens (f/1.2) fitted with a 607 ± 18 nm bandpass filter was mounted onto the camera. The camera imaged a 22 $\times$ 22 mm^2^ region of interest on the phosphor coating with a projected pixel size of 180 µm/pixel.

The lifetime-based intensity ratio method (Ojo et al. 2023; Burnford et al. 2024) was used to measure surface temperature from the phosphorescence signal. The high-speed camera operated at a frame rate of 60 kHz, with an interframe time of 400 ns and camera exposure of 16.35 µs. For each 1 kHz laser pulse, three camera frames were acquired. The first frame provided a background image (BG). The second frame (F1) imaged the first 0.7 µs of the phosphorescence decay, while the third frame (F2) imaged the remaining phosphorescence signal within the 16.35 µs exposure. Figure 3a shows these frames in relation to the phosphor decay. For each decay signal, the lifetime-based intensity ratio (IR) image was evaluated as:

2 $${\text{IR}} = \frac{{{\text{F}}1 - {\text{BG}}}}{{{\text{F}}2 - {\text{BG}}}}$$

A flat-field correction was applied to correct for spatial variation of the laser beam. A time-averaged IR image recorded at room temperature was used to provide a flat-field correction ratio image (R):

3 $${\text{R}} = \frac{{{\text{IR}}}}{{{\text{IR}}_{{294{\text{K}}}} }}$$

Calibration of R as a function of temperature was performed in a similar manner as the 0D measurements. The same phosphor-coated aluminium-block was placed on a hotplate and then removed to cool to ambient temperature. The block was equipped with a thermocouple attached to its surface. While recording the temperature from the thermocouple, phosphor images were recorded and the ratio image R was determined at select temperatures to provide a calibration curve. Figure 3d shows the calibration curve depicting the temperature dependence of the spatially averaged ratio ${\text{R}}_{\text{mean}}$. This calibration curve was used to convert $\text{R}$ to surface temperature on a pixel-by-pixel basis. Figure 3d also shows the temperature dependence of the phosphor lifetime used for 0D measurements. It is shown that ${\text{R}}_{\text{mean}}$ exhibits a strong dependence on temperature as the phosphorescence lifetime decreases with temperature due to thermal quenching. Further details and various applications of the lifetime-based intensity ratio method are described in Ojo et al. (2023), Burnford et al. (2024), Padhiary et al. (2024), and Morrisset et al. (2024).

The uncertainty of the 2D phosphor thermometry measurements is reported as the spatial standard deviation evaluated from a 37 mm^2^ region in the calibration experiments. For the range of temperatures presented in the findings in Sect. 3 (290–320 K), the uncertainty is reported as 0.6–0.7 K. This uncertainty is slightly higher than reported in our previous work which reported an uncertainty of 0.3 -0.4 K for the same temperature range (Ojo et. al. 2023). The lower uncertainty in Ojo et al. (2023) was achieved by applying a 2 × 2 binning to the phosphor images, which was not performed in the current work.

### Flame Front Imaging

Chemiluminescence images were recorded simultaneously with phosphor images to visualize the flame front in conjunction with T_wall_. A high-speed camera (VEO 710L, Phantom) equipped with a 50 mm Nikon lens (f/1.2) was used to image chemiluminescence. For 0D T_wall_ measurements, the camera directly imaged the chemiluminescence in the crevice region through the 110 mm × 60 mm area of the front window (Fig. 1b). For 2D T_wall_ measurements, the camera imaged the same ROI, but a dichroic mirror (DMLP505L, Thorlabs) was used to reflect light less than 505 nm for chemiluminescence imaging, while light above 505 nm was transmitted through the dichroic mirror to the phosphor camera (see Fig. 1c). The imaged region for chemiluminescence included the 22 × 22 mm^2^ ROI captured by the phosphor images. The chemiluminescence images had a projected pixel size of 180 µm/pixel, matching that of the phosphor images. The chemiluminescence images and phosphor images were spatially mapped in MATLAB such that both images were spatially mapped with respect to each other.

In both setups, for CH_4_-air experiments, a 433 ± 14 nm bandpass filter was used in front of the camera lens to image CH* chemiluminescence. For H_2_-air experiments, a 310 ± 10 nm bandpass filter was placed in front of the camera lens in an attempt to image OH*. However, the lean H_2_-air mixtures in combination with non-optimal detection system in the UV (e.g., 70% filter transmission, combined ~ 1% 310 nm transmission from the Nikon lens and camera spectral throughput at 310 nm) yielded weak OH* signals. Therefore, the filter was removed, which allowed the camera to detect the OH* signal (with estimated ~ 1% detection efficiency), as well as an infrared emission from vibrationally excited H_2_O at wavelengths greater than 700 nm and a weak “blue continuum” as reported by Schefer et al. (2009). Without the use of the bandpass filter and accounting for the spectral throughput of our imaging system, it is estimated that the chemiluminescence signals are mainly from the H_2_O* emissions in the infrared, while OH* and the “blue continuum” contribute to a lesser extent. The infrared emissions are captured due to the reflectance of the dichroic mirror, which opens up above 800 nm. The resulting chemiluminescence signals provide sufficient information of the global flame features, which are helpful when interpreting the T_wall_ images.

For 0D T_wall_ measurements, the chemiluminescence camera operated at a 2 kHz frame rate with an exposure time of 500 µs. For the 2D T_wall_ measurements involving the CH_4_-air experiments, the camera also operated at a 2 kHz frame rate and 500 µs exposure time, such that chemiluminescence was captured either during or after the phosphorescence. The 433 ± 14 nm bandpass filter removed most of the laser light, therefore, it was possible to excite the phosphor and capture its phosphorescence during the 500 µs exposure of the CH* image. With this timing, the phosphorescence images could be easily correlated with CH* images. For H_2_-air experiments, because a filter was not used to remove laser light, the camera was operated at a 1 kHz frame rate such that the 500 µs exposure of chemiluminescence was designated to occur 30 µs after the laser excitation to avoid capturing laser light into the chemiluminescence image. Figure 3b, c show these exposure timings relative to the phosphorescence decay. While this work did not employ the same chemiluminescence exposure timing relative to the phosphorescence decay for CH_4_ and H_2_ experiments, the flame images in both cases provided valuable information to understand the T_wall_ and flame dynamics.

## Results and Discussion

### Overview of Flame Visualization in Crevice

**Fig. 4:**
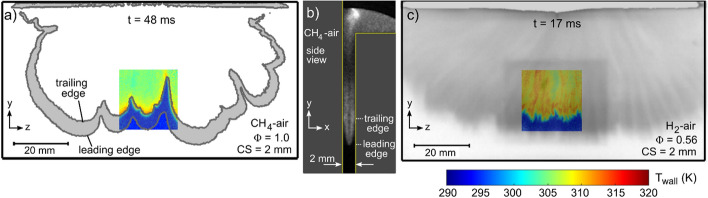
**a** CH* and corresponding T_wall_ for CH_4_-air Ф = 1 and CS = 2, **b** Orthogonal (side) view of the flame in crevice for CH_4_-air Ф = 1, CS = 2 mm, **c** chemiluminescence and corresponding T_wall_ for H_2_-air Ф = 0.56 and CS = 2 mm

Before evaluating the spatiotemporal features of T_wall_, it is first helpful to describe the general flame characteristics in the crevice for the CH_4_ and H_2_ mixtures. As the flame reaches the crevice region, it propagates into the narrow passage of the crevice. The flame traveling in the crevice emulates a closed Hele-Shaw like configuration (Al Sarraf et al. 2019). Figure 4 shows chemiluminescence images of the flame in the crevice at select timings for the CH_4_-air Φ = 1.0 and H_2_-air Φ = 0.56 mixtures. Images are shown with CS = 2 mm and the flame is shown within the entire span of the crevice to describe its overall geometry relative to the 22 × 22 mm^2^ phosphor ROI where T_wall_ is measured. The difference in flame propagation speed is evident by the timing of the images, where *t* = 0 is the start of experiment (*t* = 8 ms is start of ignition). The H_2_-flame arrives nearly three times faster to the ROI than the CH_4_-flame. The difference in flame propagation speed is also evident by the differences in the pressure curves shown in Fig. 2.

For the CH_4_-air mixture, the slow flame propagation is more conducive for chemiluminescence to resolve the detailed flame features. In the crevice, the CH_4_-flame exhibits large-scale wrinkling, which varies spatially and temporally. In Fig. 4a, the flame shown by the CH* signal appears as a thickened or elongated band. As discussed in our previous work (Ojo et al. 2022, 2023), this band is best explained when imaging in a plane orthogonal to the wall. Such an orthogonal view is shown in Fig. 4b for the CH_4_-air mixture (note that CH* images in Fig. 4a and b correspond to different experiments). The flame in Fig. 4b depicts an arrow-head shape with a leading edge centered within the crevice and trailing edges located closest to the walls. This arrow-head flame shape ultimately gives the elongated CH* band in Fig. 4a, where the leading and trailing edges are explicitly labeled. As shown in our previous work (Ojo et al. 2021, 2022, 2023), T_wall_ increases sharply at the trailing edge of the CH* signal because it is the flame’s trailing edge that is closest to the wall. Therefore, under the CH_4_-air conditions employed in this work, T_wall_ conforms to the flame’s trailing edge geometry, which is clearly seen in Fig. 4a.

For the H_2_-air Ф = 0.56 mixture, the features of the flame front are not well resolved within the chemiluminescence images. This is primarily due to the speed of the flame relative to the long camera exposure of 500 µs. Within the 500 µs exposure, the flame travels ~ 10 mm on average, which makes it challenging to capture a “still” image of the flame front. The resulting chemiluminescence image shown in Fig. 4c resolves a smeared-like flame, where the leading edge of the flame is shown further downstream of the sharp increase in wall temperature. Laser-induced fluorescence (LIF) imaging of OH radicals with 100 ns exposure time would be better suited to capture a “still” image of the flame. Unfortunately, OH-LIF was unavailable for these experiments.

Despite not capturing a “still” image of the H_2_ flame features, the chemiluminescence images for H_2_ reveal distinct features worth noting. In particular, elongated vertical streak patterns are shown in the chemiluminescence image in Fig. 4c. The corresponding T_wall_ image also shows a vertical streak pattern with intermittent high- and low-temperature streaks. These vertical streaks appear consistent with what is often referred to as “flame fingers”, which are relatively large-scale wrinkling features often present for lean premixed H_2_-flames (Fernández-Galisteo et al. 2018; Berger et al. 2019). These flame fingers can be induced by hydrodynamic instabilities (Frouzakis et al. 2015), which can be exacerbated by elevated gas pressures (Attili et al. 2021), or can be introduced by the redirection of the flame as it enters the crevice (i.e., disturbance of the flame). While it is not possible to match the spatiotemporal attributes of the flame and T_wall_ for the H_2_-air Ф = 0.56 mixture, the chemiluminescence images provide sufficient insight into the T_wall_ features resolved from phosphor thermometry.

It should also be mentioned that the T_wall_ images do not exhibit a smearing effect associated with the rapid flame propagation of H_2_ flames. The phosphorescence lifetime decay of the ScVO_4_:Bi^3+^ phosphor used in this work is less than 3 µs at room temperature, and the lifetime decreases with increasing temperature (Abram et al. 2020; Ojo et al. 2022). With a flame progression of 20 m/s on average, the flame will move 60 µm within the maximum time of the phosphorescence lifetime. This 60 µm flame movement is below the projected pixel size of 180 µm/pixel. Therefore, the phosphor images suitably capture the wall temperature when the H_2_ flame is relatively stationary.

### *Spatiotemporal Flame and T*_*wall*_* Dynamics with CS* = *2 mm*

**Fig. 5:**
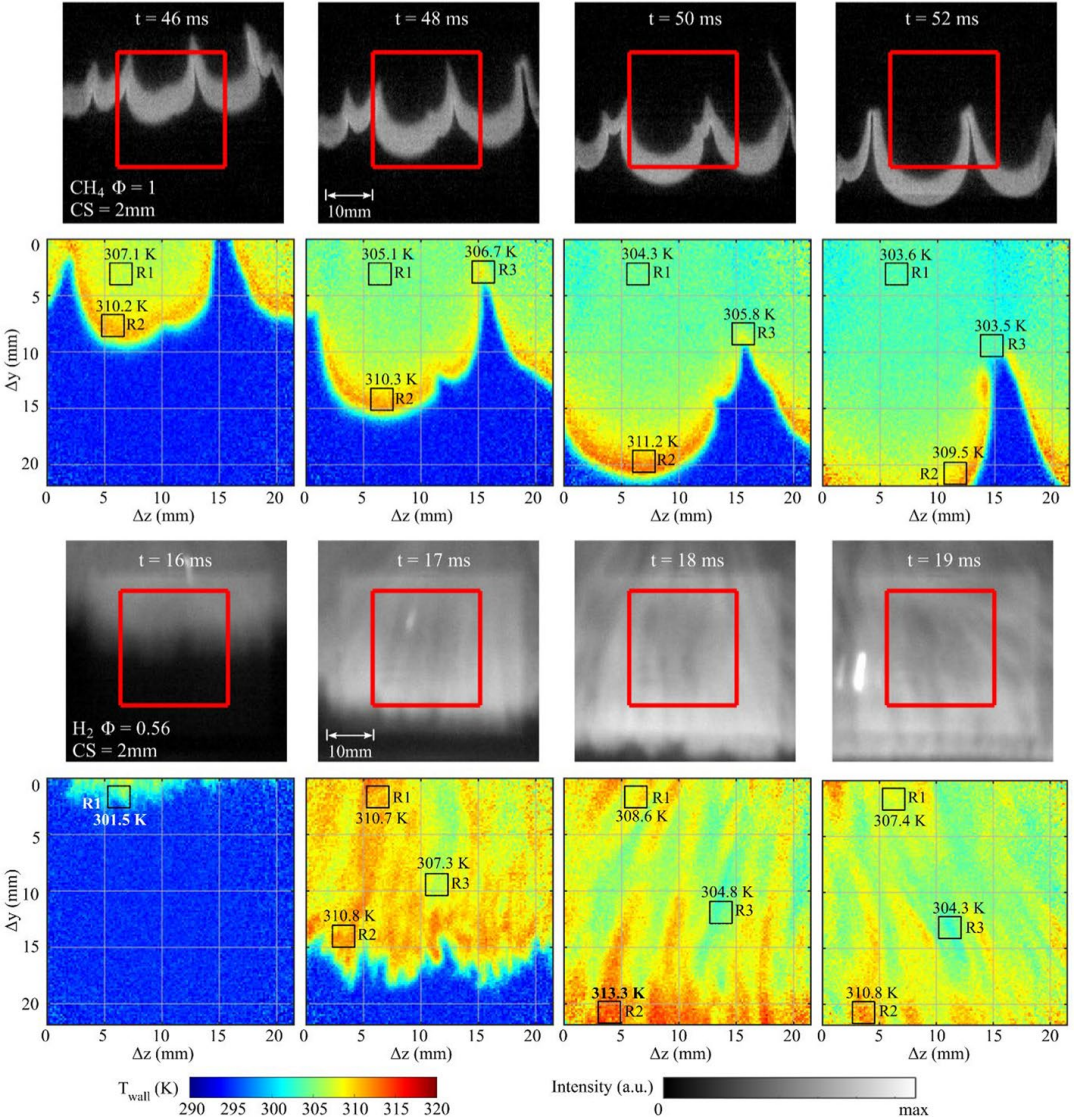
Chemiluminescence and T_wall_ image sequence for CH_4_-air Ф = 1 and H_2_-air Ф = 0.56 cases with CS = 2 mm. The CH_4_-air Φ = 1 case is shown in the top two rows, while the H_2_-air Φ = 0.56 case is shown in the bottom two rows. Rectangles report the spatially-averaged T_wall_ at a fixed location (R1), at the highest temperature region (R2), and at the lowest temperature region near the flame (R3)

This section presents temporal sequences of chemiluminescence and T_wall_ images to describe the wall temperature distributions associated with the CH_4_ and H_2_ flame behavior in the crevice. Figure 5 shows select images from 1 kHz imaging sequences for the CH_4_-air Φ = 1 and H_2_-air Φ = 0.56 cases with CS = 2 mm. Although Fig. 5 shows images from individual experiments, the flame and T_wall_ features shown are consistent with all experiments performed at these conditions. The red rectangle shown in the chemiluminescence images corresponds to the 22 × 22 mm^2^ region where T_wall_ is measured. In the T_wall_ images, spatially-averaged surface temperatures are extracted from rectangles R1, R2, and R3 (each of 2 × 2 mm^2^ area) to help quantify and compare spatial features of T_wall_ between the CH_4_ and H_2_ flames. R1 is fixed in space and represents a traditional point-wise phosphor thermometry measurement, similar to what is reported in Sect. 3.3. R2 and R3 are selectively placed to track the hottest and coldest T_wall_ locations (respectively) near the flame front. Identifying the exact location of the flame front for H_2_ cases is more challenging. For H_2_ cases, R2 and R3 are placed in hot and cold streaks, respectively. A rectangle is not shown if the particular feature is not present within the image.

In the case of CH_4_-air Φ = 1, the flame exhibits large-scale wrinkling with distinct flame crests and cusps, which are defined to be convex and concave towards the reactants, respectively. The temperature images show that T_wall_ conforms to the distinct flame geometry, including the large and fine scale wrinkling. Images show that T_wall_ is highest immediately behind the flame’s trailing edge, and monotonically decreases with increasing distance from the flame. In Fig. 5, it is also shown that T_wall_ is consistently highest behind flame crests, while flame cusps exhibit appreciably colder temperatures. Rectangles R2 and R3 track these features throughout the image sequence.

**Fig. 6:**
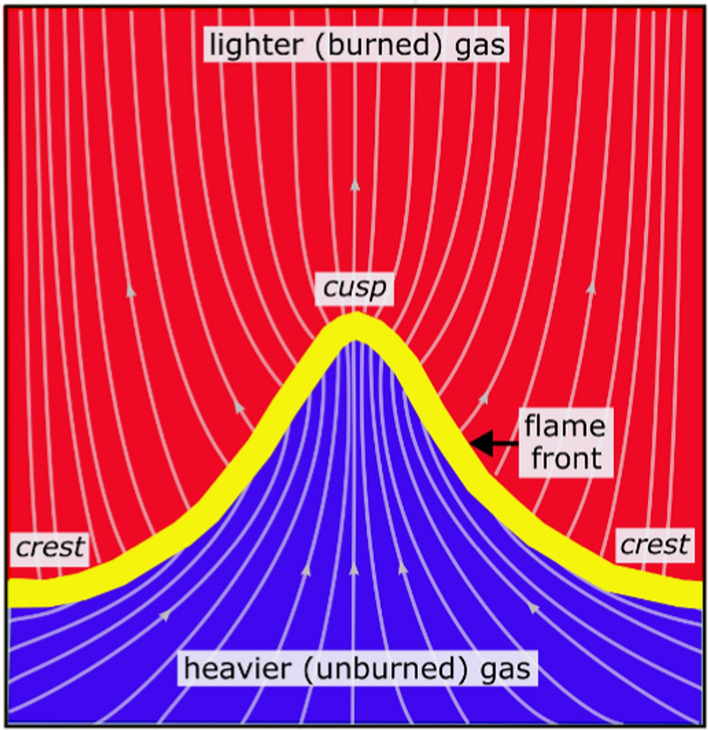
Example flow field emulated after the work of Folga et al (2017) describing the qualitative flow features associated with a cusp-like flame resulting from the Darrieus-Landau instability

The lower temperatures at flame cusps has been discussed in our previous work (Ojo et al. 2023). As discussed in that work, the effects of the hydrodynamic instability associated with a wrinkled flame front is hypothesized to cause the lower temperature associated with flame cusps. The hydrodynamic instability (or the Darrieus-Landau instability) arises from thermal expansion across the flame front (Matalon 2007). This instability often creates an intrinsic flow pattern in relation to a wrinkled flame front. Figure 6 shows an exemplar flow field emulated after the work of Folga et al. (2017), which describes the flow features of a cusp-like flame resulting from the Darrieus-Landau instability for a flame propagating into a quiescent propane-air mixture. The generic flow field in Fig. 6 is presented to highlight common features resulting from the Darrieus-Landau instability. For a wrinkled flame front, the density jump across the flame front causes the streamlines ahead of flame crest regions to diverge, reducing the velocity in that region. The flow is consequently channelled into the flame cusp regions and the flow velocity increases in this region. It is hypothesized that the accelerated flow into the cusp region can lead to the lower T_wall_ in flame cusp regions. For example, the increased unburned gas velocity is anticipated to slow the flame propagation in cusp regions. Slower flame propagation can be seen in the image sequence in Fig. 5, where the trough of the flame cusps propagates less quickly than other flame regions, making the flame cusp more pronounced (see also movie sequences in supplemental material). Longer residence times can be associated with the slower flame propagation, for which the cusp region can have more time for heat transfer at the wall. In addition, the flow velocity channelled into cusp regions can increase the strain rate at the surface of the flame cusp. The increased heat loss and strain rate can weaken the flame and lead to lower temperatures imparted onto the wall in cusp regions.

Referring back to Fig. 5, at *t* = 52 ms, T_wall_ is shown to be significantly colder at the flame cusp, almost as if a flame is not present at the trough of the cusp. Such features were also reported in Ojo et al. (2023). As the flame cusp becomes more pronounced it is suspected that the unburned gas is strongly channelled into the cusp region and this colder gas impinges onto the wall. It is suggested that this flow directly cools the wall or diverts the flame further from the wall, which results in colder T_wall_ at the trough of the flame cusp. The supplemental material shows the high-speed movie of the full image sequence, which shows further details of the flame/wall dynamics. Additional measurements and numerical simulations emulating our conditions would are needed to explore the aforementioned hypotheses further.

For the H_2_-air Ф = 0.56 case shown in Fig. 5, the T_wall_ signatures resemble a similar vertical streak pattern similar to those observed in the chemiluminescence images. As mentioned in Sect. 3.1, the flame has progressed further in the chemiluminescence images than the "temperature front" in the T_wall_ images because the phosphor images are acquired before the chemiluminescence image and the flame progation is fast (~ 20 m/s). At t = 17 ms, the flame is near the bottom of the ROI. The border where T_wall_ increases from its ambient value of ~ 293 K to a higher temperature is noticeably jagged in comparison to the CH -air flame (~ 20 m/s). Directly downstream of the jagged border, T_wall_ is comparable in magnitude to the CH_4_-air case with T_wall_ ranging from 307 – 312 K. Several hot vertical streaks are shown to extend upwards from this region. For example, a hot temperature streak extends from R2 to R1 and the temperatures in these rectangles are very similar despite having a large $\Delta y$ separation. Overall, the walled regions covered by the flame exhibit a higher *average* T_wall_ in the ROI than the CH_4_-air Φ = 1 cases. The higher average T_wall_ is a direct consequence of the faster heat release (see Fig. 2) and less time for heat to dissipate in the wall from the time it was first exposed to burned gases. For example, it takes less than 2 ms for the H_2_-air flame to travel the $\Delta y$ domain of the ROI, while it takes ~ 8 ms for the CH_4_-air flame to travel the same distance. For H_2_-air Φ = 0.56, the walled regions downstream the flame at *t* = 17 ms have just been exposed to the flame and the majority of this region exhibits temperatures above 308 K. For CH_4_-air Φ = 1, the equivalent wall region exposed to the flame and exhibiting T_wall_ in excess of 308 K corresponds to a thin 1–2 mm region directly downstream the flame front.

At *t* = 18 ms within the H_2_-air Φ = 0.56 image sequence, the leading part of the flame is beyond the phosphor ROI and the bottom of the image contains the highest temperatures across its lower boarder with R2 reaching ~ 2 K higher than that reported for the CH_4_-air case. The remainder of the ROI exhibits an alternating pattern of hot and cold vertical streaks of width between 2–3 mm. This vertical streak pattern continues at *t* = 19 ms, but overall, T_wall_ is colder due to more time for heat transfer and the streaks are shown to curve in the opposite direction as those shown at *t* = 18 ms. The change in curvature is likely due to hydrodynamic fluctuations associated with opening of the dump valve or the flame reaching the extent of the crevice region.

Overall, the H_2_-air Φ = 0.56 case exhibits wildly varying spatiotemporal flame and T_wall_ features. In the burned gas region, T_wall_ can exhibit strong variations in temperature depending on the transient nature of the hot and cold streaks. The transient spatiotemporal nature of these streaks can best be seen by the high-speed video in the supplemental material. The image sequences shown in Fig. 5 reveal that the 1 kHz imaging rate is too slow to resolve the transient T_wall_ features sufficiently for the H_2_-air Φ = 0.56 case. This is in contrast to the CH_4_-air Φ = 1 case, where the 1 kHz imaging rate resolves the transient T_wall_ features in appropriate detail.

### *Temporally-Resolved 0D T*_*wall*_* Measurements at 10 kHz*

The 1 kHz imaging rate is insufficient to resolve the temporal flame-wall dynamics for the H_2_-air Φ = 0.56 mixtures. T_wall_ images show large changes between successive measurements, which makes it difficult to understand FWI and measure the transient wall heat flux. To resolve the temporal evolution of T_wall_ in sufficient detail, experiments employing point-wise (0D) phosphor thermometry at 10 kHz were performed. As mentioned in Sect. 2.2, the 0D measurement resolves T_wall_ within a 7 mm^2^ area in the central $\Delta y$ location on the phosphor coating.

**Fig. 7:**
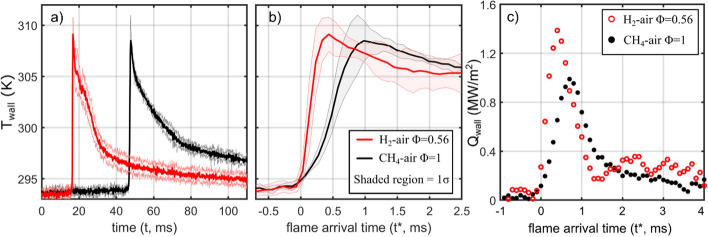
**a** Ensemble-average T_wall_ with respect to experiment duration, t = 0 refers to the start of ignition. **b** Ensemble-average T_wall_ with respect to the time the flame arrives at the measurement location (t* = 0). Ensemble-average is reported from 7 repeated experiments. The shaded region indicates one standard deviation (σ). **c** Single-experiment wall heat flux calculated from Duhamel integral. Crevice spacing is 2 mm for the results shown

Figure 7a, b shows the ensemble-average T_wall_ evolution for the CH_4_-air Φ = 1 and H_2_-air Φ = 0.56 cases with CS = 2 mm. The ensemble-average is reported from 7 experiments at each condition and the standard deviation (σ) is shown by the shaded region. In Fig. 7a, T_wall_ is reported as a function of the experiment time duration, where *t* = 0 refers to the start of experiment. Figure 7b reports the same data, but now T_wall_ is reported relative to the flame arrival time, where t* = 0 refers to the time the flame arrives at the phosphor measurement location, which is determined as the time T_wall_ rises to 10% of its maximum temperature.

In Fig. 7b, it is shown that T_wall_ increases more quickly for H_2_ than for CH_4_ cases. For H_2_, T_wall_ increases from its ambient temperature (~ 294 K) to its maximum at a heating rate of 34.6 K/ms, while the heating rate for CH_4_ is 14.5 K/ms. The faster rise in T_wall_ is expected due to the faster heat release of H_2_ as shown by the pressure curves in Fig. 2. Despite the faster temperature rise, the maximum T_wall_ reached in each case is similar. After T_wall_ reaches a maximum, the H_2_ cases are shown to exhibit larger temperature variations, which are arguably due to the spatially varying hot and cold temperature streaks in the burned gas region. As T_wall_ decreases after FWI, Fig. 7a shows that T_wall_ cools faster for H_2_ than for CH_4_. For example, T_wall_ decreases from its maximum to a near ambient value of 298 K at a rate of 0.71 K/ms for H_2_, while this cooling rate is 0.33 K/ms for CH_4_. The faster wall cooling is a result of faster gas expansion during the exhaust event. As shown in Fig. 2, the H_2_ cases exhibit a sharper pressure decrease from the higher peak pressure. The rapid gas expansion will reduce gas temperature more quickly, which promotes faster wall cooling for H_2_ cases.

The T_wall_ measurements resolved at a sufficient temporal resolution enable the calculation of the wall heat flux (Q_wall_). In this work, Q_wall_ is calculated based on the time-dependent surface temperature of a thermally semi-infinite solid. Q_wall_ is calculated assuming 1D transient heat conduction using a Duhamel integral to provide the time evolution of Q_wall_ given by (Taler and Duda 2006):

4 $$Q_{{{\text{wall}}}} \left( t \right) = \sqrt {\frac{{\rho C_{p} k}}{\pi }} \mathop \smallint \limits_{0}^{t} \frac{{dT_{{{\text{wall}}}} \left( {\overline{\tau }} \right)/d\overline{\tau })}}{{\sqrt {t - \overline{\tau }} }}d\overline{\tau }$$

In Eq. 4, the variables$\rho$,${C}_{p}$, and $k$ are the density, specific heat capacity, and thermal conductivity of the solid material, respectively, while $t$ is time and $\overline{\tau }$ is the time variable of integration. It is often appropriate to consider the physical properties of the phosphor when calculating the wall heat flux (Witkowski and Rothamer 2023). The density and heat capacity of the host material ScVO_4_ are $\rho$ = 3650 kg/m^3^ (Persson 2014) and ${C}_{p}$ = 693 J/kgK (Gavrichev et al. 2012). The thermal conductivity of ScVO_4_ was not found in the literature. Thermal conductivity values for phosphors such as HoVO_4_ and Pr:YAG have been reported in the literature as $k$ = 2 W/mK (Kondrat’eva et al. 2021; Witkowski and Rothamer 2023). Given that ScVO_4_ and HoVO_4_ belong to the same group of orthovanadate compounds with similar tetrahedral zircon-type structure, and the fact that phosphors of different compounds can yield similar conductivity values, the value of $k$ = 2 W/mK is used to calculate Q_wall_ in Eq. 4.

Figure 7c shows temporally resolved Q_wall_ traces for individual experiments with H_2_-air Φ = 0.56 and CH_4_-air Φ = 1. The H_2_-air Φ = 0.56 case exhibits a faster rise in Q_wall_ and reaches a higher Q_wall,max_ value of 1.39 MW/m^2^, while the CH_4_-air Φ = 1 case reaches a Q_wall,max_ of 0.99 MW/m^2^. The difference in Q_wall,max_ is nearly directly proportional to the difference in flame power (Q_Σ_) reported for the two mixtures (see Table 1). The maximum non-dimensional heat flux (Q_wall,max_/Q_Σ_) is 0.22 and 0.24 for the H_2_-air Φ = 0.56 and CH_4_-air Φ = 1 cases, respectively. The similar Q_wall,max_/Q_Σ_ values indicates that the wall heat loss is similar for each flame in these experiments. These trends are qualitatively similar for all experiments performed in this work under these conditions. It is also worth noting that the measurements for H_2_-air Φ = 0.56 are still likely under-resolved even at the 10 kHz repetition rate. As shown in Fig. 7c, only a few datapoints are resolved during the Q_wall_ rise for H_2_-air Φ = 0.56. As such, Q_wall,max_ may be under-reported in Fig. 7c. Nonetheless, the 10 kHz data provide a suitable understanding of the transient T_wall_ response, which can be used in combination with the 2D T_wall_ images.

### Effect of Surface Area to Volume (SA/V) on Heat Transfer

Although the 1 kHz repetition rate of the 2D measurements is ill-suited to resolve the fine temporal progression of the flame and T_wall_ for the H_2_-air Φ = 0.56 cases, access to 2D information provides useful insight into the FWI and its associated heat transfer to the wall. This section presents temporal sequences of flame and T_wall_ images at a smaller crevice spacing of 1.2 mm to demonstrate the increased wall heat loss due to a larger surface area to volume ratio. Ojo et al. (2022) evaluated T_wall_ as a function of CS for CH_4_ flames using 0D measurements at 6 kHz. This work complements that analysis by describing the effect of heat transfer on the *spatial* T_wall_ distribution for both CH_4_ and H_2_ flames. The surface area to volume ratio (*SA/V*) for CS = 1.2 mm is 1.69 mm^−1^. The images shown in this section will be compared to those presented in Sect. 3.2 with CS = 2 mm (*SA/V* = 1.02 mm^−1^).

**Fig. 8:**
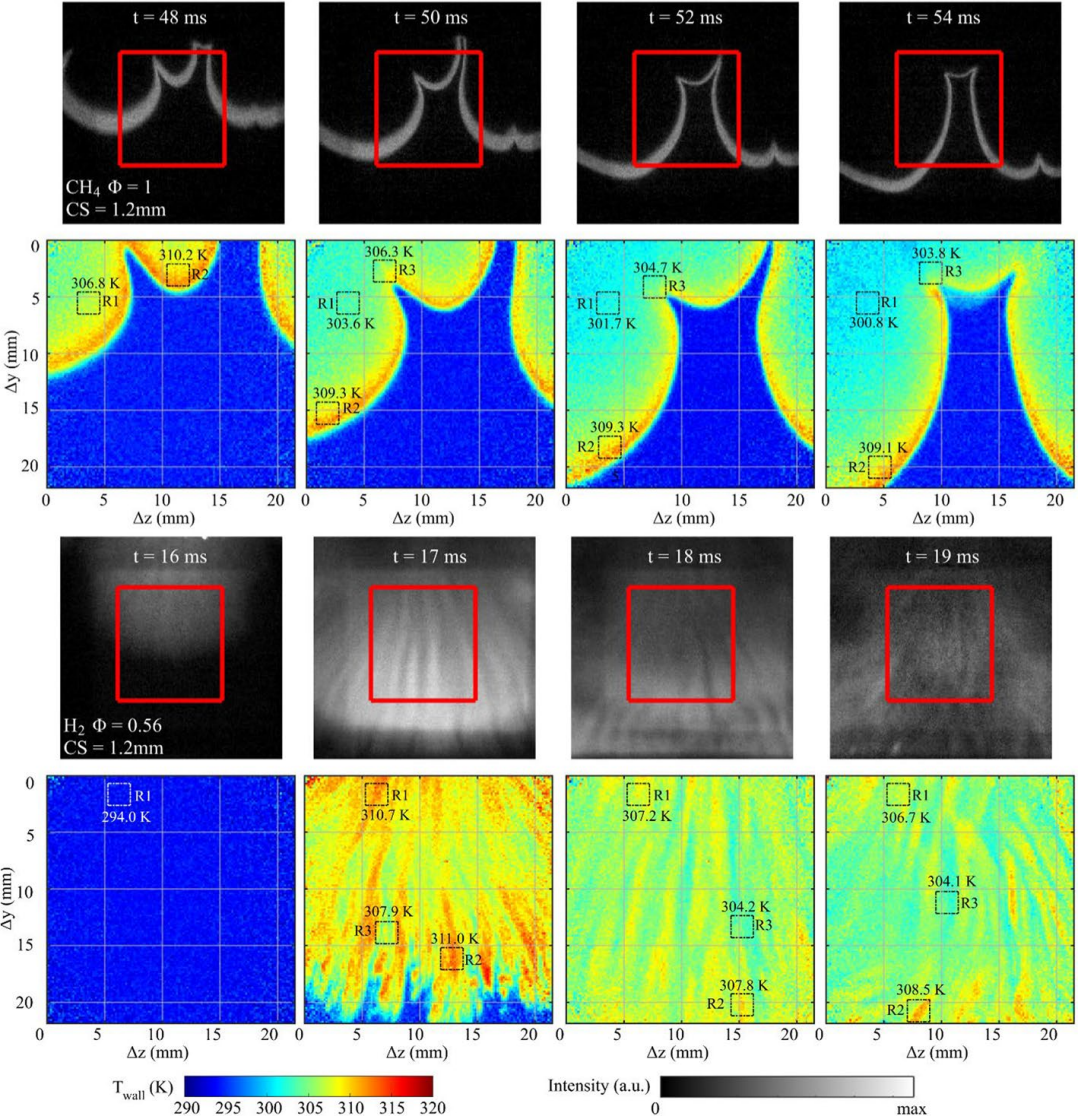
Chemiluminescence and T_wall_ image sequences for CH_4_-air Ф = 1 and H_2_-air Ф = 0.56 cases with CS = 1.2 mm. The top two rows show CH_4_-air Φ = 1, while the bottom two rows show H_2_-air Φ = 0.56. Rectangles report the spatially-averaged T_wall_ at a fixed location (R1), at the highest temperature region (R2), and at the lowest temperature region near the flame (R3). The CH_4_ sequence is shown 2 ms later than the CS = 2 mm case in Fig. 5 because the flame arrives ~ 2 ms later in the ROI

Figure 8 shows the temporal sequence of the flame and T_wall_ for CH_4_-air Φ = 1 and H_2_-air Φ = 0.56 cases with CS = 1.2 mm. It is noted that CH* signals for the CH_4_-air flame are thinner in several regions, particularly in regions that pertain to flame cusps. The thinner CH* regions occur when the flame progression slows and the distance between the flame’s leading edge and trailing edge (see Fig. 4b) is small. These features have been discussed in detail in Ojo et al. (2022, 2023).

Overall, the spatial features of the flame and T_wall_ for CS = 1.2 mm in Fig. 8 are qualitatively similar to those shown for CS = 2 mm in Fig. 5. For CH_4_, the flame exhibits large-scale wrinkling and T_wall_ is highest at the trailing edge of the flame. In addition, T_wall_ is consistently higher for flame crest regions compared to flame cusp regions as was shown in Fig. 5 for the CH_4_ flame. However, for CS = 1.2 mm, the temperatures in R2 and R3 regions are often 1–3 K lower than the CS = 2 mm case. In addition, wall temperatures downstream of the flame front are shown to cool more rapidly for CS = 1.2 mm than for CS = 2 mm. For example, within the image sequence shown, T_wall_ reported within R1 cools at a rate of 1.13 K/ms for CS = 1.2 mm, while for CS = 2 mm, the wall cools at a rate of 0.58 K/ms. That is, for an increase in *SA/V* of ~ 65%, the cooling rate is shown to increase by ~ 95%. The disproportionate increase in heat loss with *SA/V* is consistent with Ojo et al. (2022), where cooling rates were measured using 0D phosphor thermometry for CS ranging from 3.5 mm to 1.2 mm.

For the H_2_-air Ф = 0.56 image sequence with CS = 1.2 mm, the T_wall_ images exhibit hot and cold temperature streaks, which emulate the vertical streak pattern in the chemiluminescence images. The size of the temperature streaks is narrower for CS = 1.2 mm than for CS = 2 mm, which may be a consequence of the narrower CS. As the flame travels through the ROI at *t* = 17 ms, the wall temperature distribution is similar to that shown for CS = 2 mm with the temperatures in the hot and cold streaks being comparable in value. Again, the walled regions covered by the flame have a higher *average* T_wall_ in the ROI compared to the CH_4_-air Ф = 1 images, which is a result of the faster heat release and less time for heat to dissipate in the wall for H_2_-air Ф = 0.56. After the flame travels past the ROI, the increased wall heat loss due to the smaller CS becomes more apparent. For example, T_wall_ exhibits lower overall temperatures and the highest temperatures are 3–4 K colder than those at CS = 2 mm.

**Fig. 9:**
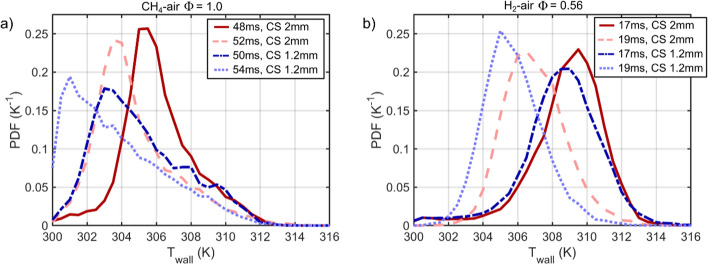
PDFs of T_wall_ at select times for the image sequences shown in Fig. 5 and 8. PDFs for CH_4_-air Ф = 1.0 are offset by 2 ms for the crevice spacings because the flame arrives ~ 2 ms later into the ROI for CS = 1.2 mm due to the flame experiencing greater resistance traveling into the narrower CS

The greater cooling effect at larger *SA/V* (i.e., narrower CS) is described further in Fig. 9, which shows PDF distributions of T_wall_ at select timings within image sequences of Figs. 5 and 8. The PDFs evaluate temperatures above 300 K to isolate wall locations that have been heated by the flame.

For the CH_4_-air Φ = 1 mixtures in Fig. 9a, PDFs are extracted when the flame is approximately half-way into the ROI. This timing corresponds to *t* = 48 ms for the CS = 2 mm case and *t* = 50 ms for the CS = 1.2 mm case. The slightly later arrival of the flame occurs for CS = 1.2 mm because the flame experiences more resistance penetrating into the narrower CS as described in Ojo et al. (2022). For the PDFs at *t* = 48 ms and 50 ms, T_wall_ extends up to 312 K and the distributions from 309 to 312 K are very similar for each CS. These temperatures represent the higher temperatures in close proximity to the flame’s trailing edge. Temperatures below 309 K represent regions further away from the flame front where temperature dissipates. The PDFs at *t* = 48 ms and 50 ms exhibit large differences below 309 K, where CS = 1.2 mm shows a substantial shift towards colder T_wall_, which demonstrates the greater cooling associated with higher *SA/V*. The PDFs 4 ms later for each CS show a shift towards colder temperatures, which is largely due to flame regions exiting the imaging region. The greater cooling effect continues at these later timings with PDFs at *t* = 52 ms and 54 ms exhibiting a larger shift towards colder temperatures, particularly for CS = 1.2 mm.

For the H_2_-air Φ = 0.56 cases shown in Fig. 9b, the PDFs at *t* = 17 ms exhibit a very similar distribution for CS = 1.2 and 2 mm, where only a mild shift towards colder temperatures exists for CS = 1.2 mm. The T_wall_ distribution at *t* = 17 ms is similar at both CSs despite the difference in *SA/V* because the heat imparted occurs very rapidly due to the fast flame propagation, and little time has progressed for heat transfer to have a strong effect on T_wall_ for each CS. As more time progresses, the wall cools for both cases, but the cooling is greater for CS = 1.2 mm. The PDF for CS = 1.2 mm shows a 1-2 K shift towards colder temperatures revealing the faster cooling rate for the narrower *SA/V*.

The qualitative trends described in Fig. 9 are consistent for other image timings within the temporal sequence. However, in order to provide a fair comparison between each CS for a given fuel, the flame should penetrate a similar distance into the field of view so that a similar area downstream the flame is evaluated for each CS.

### *FWI Dynamics Associated with Thermodiffusive Instabilities of Lean H*_*2*_* Flames*

The 1 kHz recording rate was too slow to capture temporally correlated T_wall_ images for the H_2_-air Ф = 0.56 mixtures presented in Sects. 3.2 and 3.4. To slow the flame progression, a leaner H_2_-air mixture with Ф = 0.45 was used for which the 1 kHz repetition rate was sufficient to capture a temporally correlated progression of T_wall_ imposed by the flame. At these lean mixtures, the flame is also highly susceptible to exhibit thermodiffusive instabilities, which will alter the flame dynamics (Frouzakis et al. 2015, Berger et al. 2019, Howarth and Aspden 2022). The thermodiffusive instability is a peculiar feature of lean H_2_ mixtures with low Lewis numbers (Le), where preferential diffusion of H_2_ leads to variations of the local equivalence ratio along the flame front. When this occurs, hydrogen diffuses into hotter regions, where the local reaction rate increases and the flame can reach super adiabatic temperatures (i.e., temperatures above the adiabatic temperature at the global equivalence ratio) (Frouzakis et al. 2015; Howarth et al. 2023). This preferential diffusion can therefore cause concern of high heat fluxes imposed onto surfaces.

This section presents image sequences for H_2_-air mixtures with Ф = 0.45 to study the T_wall_ features and the consequential wall heat fluxes associated with thermodiffusive instabilities of lean H_2_-air flames. Experiments were conducted with CS = 1.2 mm, which aided in slowing the flame progression in the crevice. While thermodiffusive instabilities are studied for the H_2_-air Ф = 0.45 mixture, it should be emphasized that thermodiffusive instabilities may also occur for H_2_-air Ф = 0.56 cases. The Lewis numbers for both cases are low, and thermodiffusive instabilities may develop in both conditions. However, the fact that H_2_-air Ф = 0.56 cases provide a single image where the leading flame is detected in the ROI per experiment makes it very challenging to obtain definitive evidence of these unstable flame features and their influence on wall heat transfer. The slower propagation speed associated with the H_2_-air Ф = 0.45 CS = 1.2 mm operation allows us to visualize the development of a low Le flame in a more appropriate manner and record its unique heat signatures on the wall.

**Fig. 10:**
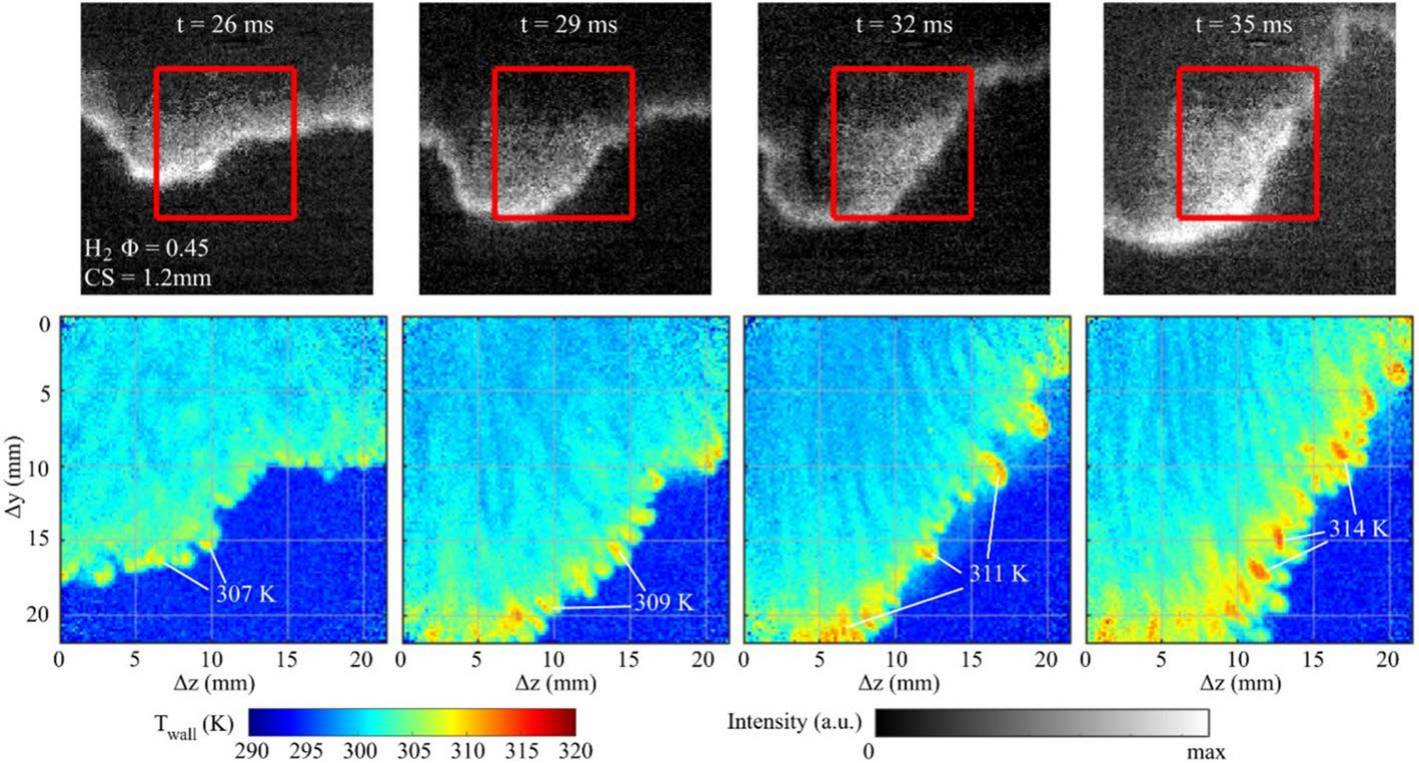
Spatiotemporal sequence of chemiluminescence and T_wall_ images capturing the wall temperature features for a flame undergoing thermodiffusive instabilities for H_2_-air Φ = 0.45 CS = 1.2 mm

Figure 10 shows chemiluminescence and T_wall_ images at select times as the flame progresses through the crevice region for a single experiment with H_2_-air Ф = 0.45. Due to the leaner mixture, the chemiluminescence has a low signal-to-noise ratio (SNR). Despite the lower SNR, it is possible to visualize the leading flame front location, while the corresponding T_wall_ images from phosphor thermometry provide insight into the geometrical flame features near the wall. In contrast to the H_2_-air Ф = 0.56 cases, the chemiluminescence and T_wall_ images do not reveal long finger-like flame structures, but instead the T_wall_ images show small cellular-like temperature structures at the leading “temperature front”. These cellular-like temperature structures take time to develop as the flame penetrates into the crevice region. At *t* = 26 ms, the cellular structures are relatively small in the T_wall_ image with temperatures reaching as high as 307 K within the cellular region. As time progresses, these cellular structures become more pronounced and the wall temperatures associated with these structures increase, reaching as high as 314 K. Although these high temperature regions occupy small regions on the wall, it is remarkable that T_wall_ reaches values as high as the CH_4_-air Ф = 1.0 and H_2_-air Ф = 0.56 CS = 1.2 mm cases, despite having a flame power that is 2.5 to 3.6 times lower, respectfully.

The cellular structures seen in the T_wall_ images in Fig. 10 are congruent with trademark features of a thermodiffusively unstable flame (Yu et al. 2017; Berger et al. 2019, 2022; Howarth and Aspden 2022; Howarth et al. 2023). Given the fact that T_wall_ images can closely resemble the near-wall flame features, it is argued that the spatial features of T_wall_ are qualitatively similar to the near-wall flame distribution as the flame becomes thermodiffusively unstable. The regions of high temperature are considered to be flamelets with locally richer H_2_ concentrations and higher flame temperatures. These flamelets, while being hotter, will also burn closer to the wall due to their higher flame speed. Both of these aspects will contribute to the higher wall temperatures. This description is qualitatively similar to DNS findings from Ghai et al. (2023), where it was reported that positively curved flame regions of low Le flames yield smaller flame quenching distances, which were a result of locally richer and hotter flame structures that could withstand greater heat loss before quenching.

**Fig. 11:**
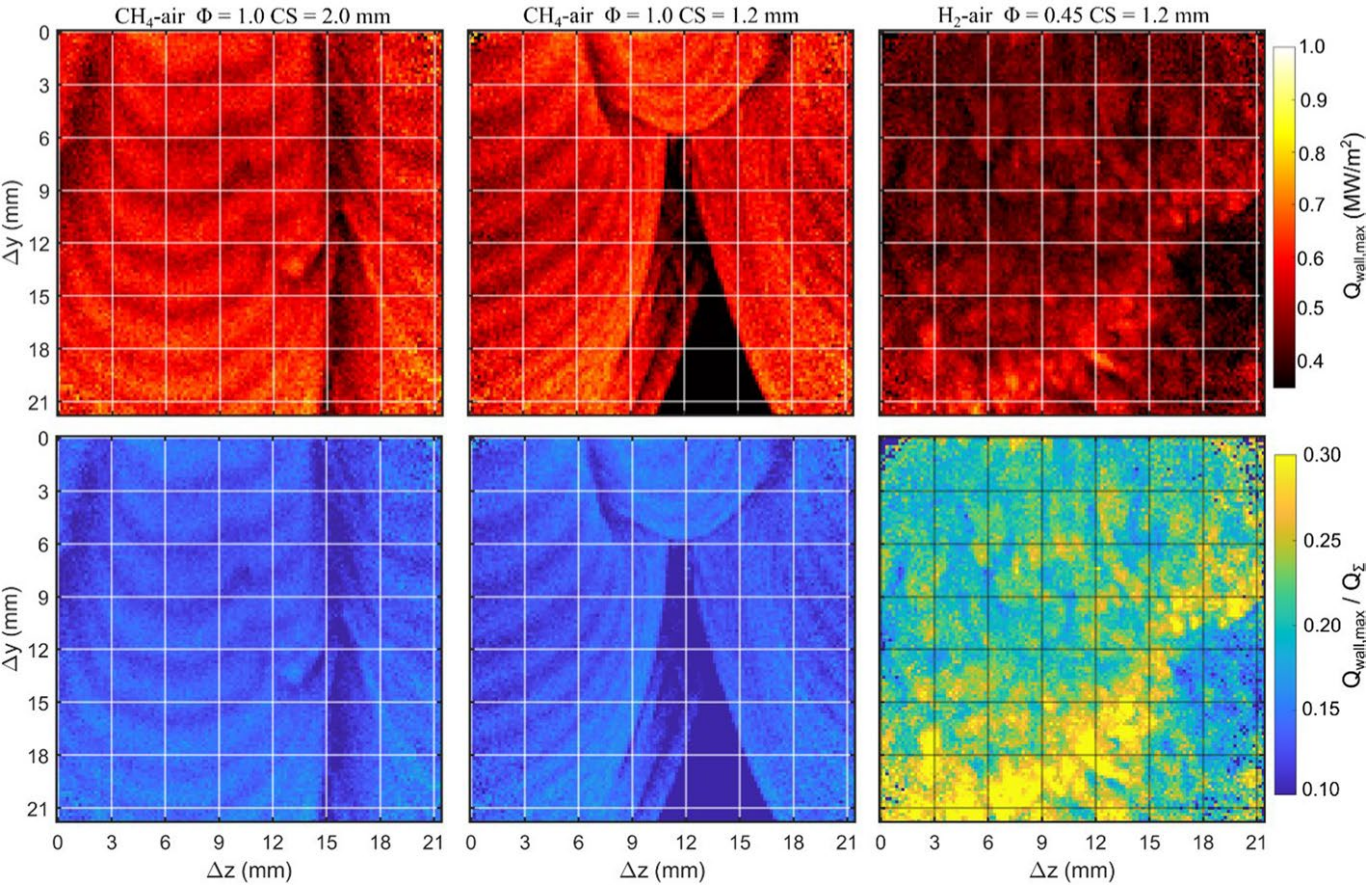
Top: Q_wall,max_ spatial distribution for the image sequences associated with CH_4_-air Ф = 1 and H_2_-air Ф = 0.45 cases shown in Figs. 5, 8, and 10. Bottom: Corresponding normalized heat flux (Q_wall,max_/Q_Σ_) for each case

With the increasing demand for hydrogen in combustion-based applications, it is important to understand the implications of the thermodiffusive flame instabilities on the wall heat flux. Using the 2D T_wall_ images, which are now sufficiently resolved temporally, Q_wall_ is calculated according to Eq. 4 at each image pixel. Q_wall,max_ is calculated using a 3-point temporal moving average to reduce measurement noise. Figure 11 (top row) reports the maximum wall heat flux (Q_wall,max_) recorded at each pixel as the flame travels through the ROI. Q_wall,max_ is shown for the full image sequences corresponding to CH_4_-air Φ = 1 with CS = 2.0 mm, CH_4_-air Φ = 1 with CS = 1.2 mm, and H_2_-air Φ = 0.45 with CS = 1.2 mm. The bottom row of Fig. 11 reports the maximum heat flux in its non-dimensional form (Q_wall,max_/Q_Σ_), where Q_Σ_ is the average flame power reported in Table 1. Q_wall,max_/Q_Σ_ provides a measure of the energy transferred to the wall relative to the theoretical available energy of the flame at the global mixture conditions. Although mixture stratification is expected when thermodiffusive instabilities occur, Q_Σ_ associated with the global mixture condition is used for normalization, which helps emphasize the deviation resulting from thermodiffusive instabilities.

The Q_wall,max_ images in Fig. 11 describe the spatially distributed wall heat flux associated with distinct flame features. The CH_4_-air cases reveal the Q_wall,max_ relative to the large-scale flame wrinkling. For example, the CH_4_-air Ф = 1 CS = 2.0 mm image shows the trajectory of two flame crests and two flame cusp regions as the flame travels vertically downwards. The Q_wall,max_ image for CH_4_-air Ф = 1 CS = 1.2 mm shows the dynamics of two flame crests merging and the formation of a large flame cusp in the center of the ROI. The trajectories of flame crest regions consistently exhibit the highest Q_wall,max_ reported in the image (~ 0.75 MW/m^2^), resulting from higher T_wall_ associated with flame crests (see Figs. 5 and 8). The trajectories of flame cusp regions exhibit Q_wall,max_ values that are approximately half of crest regions (0.4–0.5 MW/m^2^), which result from the lower T_wall_ in flame cusp regions. The maximum Q_wall_ values reported in Fig. 11 are ~ 25% lower than those reported in Fig. 7, which is a result of the lower temporal resolution of 1 kHz. Although Q_wall,max_ values in Fig. 11 are under-resolved, the images provide a useful spatial comparison between the different operating conditions. For the CH_4_-air Ф = 1 cases, both crevice spacings exhibit qualitatively similar heat flux distribution. The region of zero Q_wall,max_ at the bottom of the ROI for the CH_4_-air Ф = 1 CS = 1.2 mm case is a result of the flame not progressing over this area before the majority of the gas is evacuated during the exhaust phase.

The Q_wall,max_ image for the H_2_-air Ф = 0.45 shows the heat flux associated with the smaller-scale cellular flame structures. Q_wall,max_ is much lower over the majority of the ROI, except in the regions where flame instabilities result in locally high T_wall_ values. In these regions, Q_wall,max_ reaches the same magnitude as the CH_4_-air Ф = 1 cases, which is noteworthy given the lower global flame power for H_2_-air Ф = 0.45. The bottom row of Fig. 11 shows the normalized heat flux, which emphasizes the stark contrast in Q_wall_ relative to the available global flame power between the CH_4_-air Ф = 1 and H_2_-air Ф = 0.45 cases. For the CH_4_-air Ф = 1 mixture, Q_wall,max_/Q_Σ_ reach values up to 0.17 and is consistent for each CS. However, for the H_2_-air Ф = 0.45 mixture, the majority of the ROI exhibit Q_wall,max_/Q_Σ_ values above 0.2 (i.e., above the highest value reported for CH_4_-air Ф = 1), and the maximum Q_wall,max_/Q_Σ_ values reach 0.43. As mentioned previously, the Q_wall,max_/Q_Σ_ values reported are anticipated to be under-estimated due to the lower temporal resolution of 1 kHz, but this underestimation is expected to be the same for each case.

From the evaluation of Fig. 11, it is clear that the overall Q_wall_ imposed onto the wall is greater for CH_4_-air Ф = 1 than for H_2_-air Ф = 0.45.^1^ For CH_4_-air Ф = 1, nearly all wall regions exposed by the flame reach a value between 0.65 and 0.75 MW/m^2^, while for H_2_-air Ф = 0.45, only a small region where burning is most intense reaches such values. However, it should be mentioned that the experimental conditions employed in this work are modest in comparison to technically relevant environments that would employ lean hydrogen mixtures. For example, in this work, there is a limited amount of time for the thermodiffusive instabilities to develop within the chamber. Evidence of the thermodiffusive instabilities exists for a 10–15 ms time period in these experiments. The thermodiffusive instabilities appear to reach their most intense stage around *t* = 35 ms, where T_wall_ and Q_wall_ reach their peak. At *t* = 37 ms, the chamber pressure is already decreasing rapidly as gases are evacuated during the exhaust phase (see Fig. 2). The decreasing pressure induces gas expansion, which reduces heat release and weakens the thermodiffusive instabilities. Consequently, T_wall_ and Q_wall_ reduce substantially after *t* = 35 ms.

In the experiments, if the chamber pressure was maintained, it is reasonable to anticipate that the thermodiffusive instabilities would persist and that Q_wall,max_ values could occupy a larger region on the wall. Q_wall,max_ values may even exceed those reported if the instabilities had more time to develop. This scenario may exist in gas turbine engines, where a flame burns more continuously. In reciprocating engines, the timescales of pressure rise/decay are comparable or faster than those in the present study. However, the timescales of flame development would be much faster at the higher pressures and temperatures achieved in an IC engine. Therefore, for lean burn H_2_ IC engines, thermodiffusive instabilities may require less time to develop to a mature stage, and may influence heat fluxes on engine surfaces.

Although the modest operating conditions employed in this work do not allow us to evaluate the impact of thermodiffusive instabilities on FWI and wall heat flux for all scenarios, the findings clearly demonstrate the ability of thermodiffusive instabilities to impose heat fluxes well beyond their expected value onto surfaces. This information is important when considering thermal fatigue and the cooling processes required in next-generation hydrogen applications. The experimental measurements presented are intended to help guide numerical simulation work studying FWI and hydrogen fuels. The presented measurements, as well as our those presented in our previous work in the FVC provide a valuable database of transient FWI and heat transfer processes (Escofet-Martin et al. 2021; Ojo et al. 2021, 2022, 2023). These measurements include gas- and solid-phase temperature measurements, and are available for interested modelling partners.

## Conclusions

Phosphor thermometry was used to measure the spatiotemporal distribution of wall temperature (T_wall_) resulting from flame-wall interactions of lean H_2_-air flames and stoichiometric CH_4_-air flames. Experiments were performed in a two-walled crevice passage in an optically accessible fixed volume chamber. The phosphor ScVO_4_:Bi^3+^ was used, which offers high temperature sensitivity and measurement precision. The lifetime-based intensity ratio method was used to image T_wall_ in a 22 × 22 mm^2^ wall region with 180 µm/pixel resolution at a repetition rate of 1 kHz. Point-wise (0D) phosphor thermometry was also performed at 10 kHz using the lifetime method to resolve the evolution of T_wall_ and transient wall heat flux (Q_wall_) with better temporal precision. Chemiluminescence imaging was performed simultaneously with T_wall_ measurements to correlate the spatiotemporal heat signatures measured on the wall with their corresponding flame features.

Experiments were first performed for CH_4_-air Ф = 1 and H_2_-air Ф = 0.56 mixtures with a crevice spacing (CS) of 2 mm. The CH_4_ flames exhibit large scale wrinkling for which T_wall_ conforms remarkably well to the flame geometry. T_wall_ images reveal that flame crest regions yield higher T_wall_ and wall heat flux (Q_wall_) than flame cusp regions. The lower T_wall_ in flame cusp regions is argued to be caused by the hydrodynamic instability that can strain the flame and lead to longer residence times. These features will weaken the heat signatures of the flame imposed on the wall. For H_2_-air Φ = 0.56, the flame exhibited elongated finger-like structures, which yielded a vertically alternating high and low temperature streak pattern within the T_wall_ images. Due to the faster heat release for the H_2_ flame, a larger region of the wall was exposed to the flame in a shorter time duration. With less time for the heat to dissipate in the wall, a larger fraction of the region of interest exhibited elevated wall temperatures (e.g., T_wall_ > 308 K). For the slower CH_4_-air flames, these elevated temperature regions are restricted to a 1–2 mm region downstream of the flame front, as more time elapses allowing heat to dissipate beyond this region. The underlying differences in T_wall_ distribution, flame morphology, and flame propagation between CH_4_-air and lean H_2_-air mixtures are attributed to the differences in their Lewis numbers.

Experiments were performed with a smaller CS of 1.2 mm and compared to CS = 2 mm to study the wall heat transfer associated with larger surface are to volume ratios (*SA/V*). The spatiotemporal flame and T_wall_ features were in qualitative agreement between CS = 1.2 mm and 2 mm, but a clear shift towards colder T_wall_ existed for the smaller crevice spacing. The wall cooling rate was disproportionally larger than the increase in *SA/V*, which demonstrated the substantial wall heat loss associated with higher *SA/V*.

Leaner H_2_-air mixtures with Ф = 0.45 were employed with CS = 1.2 mm where the flame progression was slow enough to be captured by the 1 kHz repetition rate of the phosphor measurements. The 2D T_wall_ images captured the intrinsic FWI features of a thermodiffusively unstable flame as it progressed through the imaged region. The temperature front exhibited strong cellular structures, which grew in size and increased in temperature as the flame progressed in the crevice. These instabilities imposed wall temperatures and wall heat fluxes as high as those seen for flames with 2–3 times greater flame power. While these flame instabilities occur within a small space/time domain in this study, they demonstrate the unique ability to impose appreciable heat flux on surfaces. Such aspects are important to consider in terms of surface durability and required cooling demand for lean hydrogen flame applications.

## Supplementary Information

Below is the link to the electronic supplementary material.

Supplemental video of wall temperature and chemiluminescence sequence for the CH4-air Φ = 1.0 mixture and CS = 2 mm conditions shown in Fig. 5

Supplemental video of wall temperature and chemiluminescence sequence for the H2-air Φ = 0.56 mixture and CS = 2 mm conditions shown in Fig. 5

Supplemental video of wall temperature and chemiluminescence sequence for the CH4-air Φ = 1.0 mixture and CS = 1.2 mm conditions shown in Fig. 8

Supplemental video of wall temperature and chemiluminescence sequence for the H2-air Φ = 0.56 mixture and CS = 1.2 mm conditions shown in Fig. 8

Supplemental video of wall temperature and chemiluminescence sequence for the H2-air Φ = 0.45 mixture and CS = 1.2 mm conditions shown in Fig. 10

## Data Availability

Data will be made available upon request.

## Footnotes

1 Note that H_2_-air Ф = 0.56 mixture explored in this work impose higher wall heat fluxes than the CH_4_-air Ф = 1 mixture (see Fig. 7c). However, it was not possible to resolve Q_wall_ from the 2D images for H_2_-air Ф = 0.56, due to the poorer temporal resolution associated with the faster flame propagation. H_2_-air Φ = 0.56 cases are therefore not considered in this discussion.

## Declarations

### Supplementary Information

Videos of the T_wall_ and chemiluminescence image sequences presented in this work are provided as supplemental material.
